# Autologous dermis reinforcement in severe rectus diastasis with cutaneous excess: A case report

**DOI:** 10.1016/j.jpra.2026.05.046

**Published:** 2026-06-04

**Authors:** Jérôme Martineau, Aude Lehnen, Daniel F. Kalbermatten, Jonathan Douissard, Lucille Nichols

**Affiliations:** a Department of Plastic, Reconstructive, and Aesthetic Surgery, Geneva University Hospitals, Geneva University, Geneva, Switzerland; b Department of Visceral Surgery, Geneva University Hospital, Geneva University, Geneva, Switzerland

**Keywords:** Giant diastasis, Rectus diastasis, Autologous dermal mesh, Hernia, Abdominoplasty

## Abstract

Severe rectus diastasis with ventral hernia is typically reinforced with synthetic mesh, although prosthetic materials carry risks of infection and other complications. Autologous dermis harvested during abdominoplasty offers a biologic alternative. We report the case of a 36-year-old multiparous woman with giant rectus diastasis (15 cm intraoperatively; 19 cm on computed tomography) and an umbilical hernia associated with marked skin excess. She underwent abdominoplasty with placement of an autologous dermal graft in a sublay position to reinforce fascial repair. One week postoperatively, umbilical necrosis required debridement and local-flap reconstruction; subsequent recovery was uneventful. At one year, no recurrence was detected clinically or by ultrasound, and the patient reported high functional and aesthetic satisfaction.

This case demonstrates that autologous dermal reinforcement can provide durable abdominal wall support even in very large defects while avoiding prosthetic material. The absence of mesh was advantageous in the setting of umbilical necrosis, reducing the risk of mesh infection. Autologous dermis during abdominoplasty is therefore an efficient and cost-effective option for selected patients with severe rectus diastasis.

## Introduction

Reconstruction of large rectus diastasis (RD), often associated with ventral hernias, is commonly performed using synthetic mesh.^1^ However, autologous deepithelialized dermis presents a promising alternative, particularly in young patients or cases where foreign body implantation is contraindicated. Case series and reports have demonstrated the feasibility of this approach, yet data on its application in large RD exceeding 10 cm remain scarce.^1^^–^^3^ Despite its potential, this technique remains underutilized. Complications include seroma, hematoma and postoperative pain, consistent with those observed with synthetic mesh repair.^1^, ^2^, ^3^ We present a case illustrating its application and key surgical steps.

## Case presentation

**Fig. 1:**
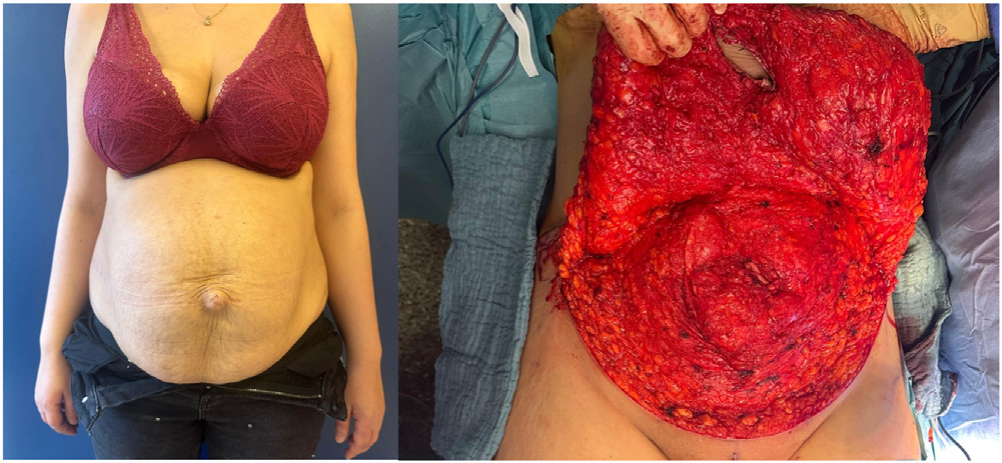
Preoperative (left) and intra-operative view (right), showing the severe RD and associated umbilical hernia.

A 36-year-old multiparous woman, with a body mass index of 27.7 kg/m^2^, one prior caesarian section in 2019 and no significant comorbidities, presented with a severe RD, measuring 15 cm at rest and expanding to 25 cm during the Valsalva maneuver, associated with an umbilical hernia, both confirmed by a computed tomography (CT). These findings were accompanied by significant abdominal skin excess following three pregnancies and postpartum weight fluctuations (Fig. 1). She reported core weakness, lower back pain, and severe body image dissatisfaction.

**Fig. 2:**
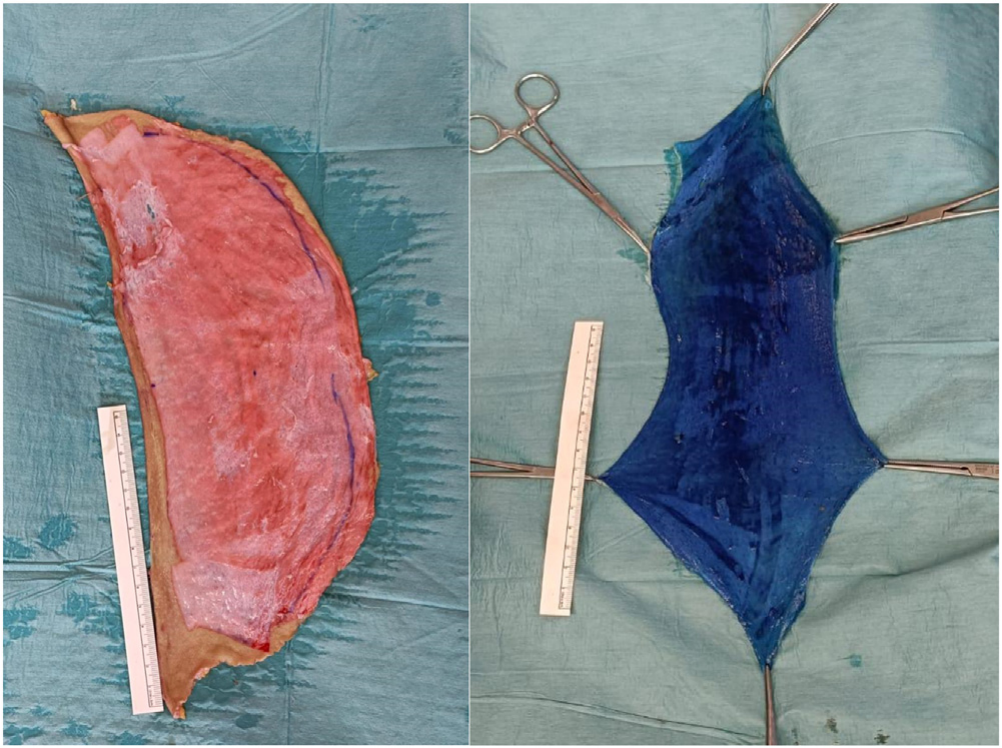
Intra-operative view: abdominal dermal autologous mesh before (left) and after (right) methylene blue dye application.

**Fig. 3:**
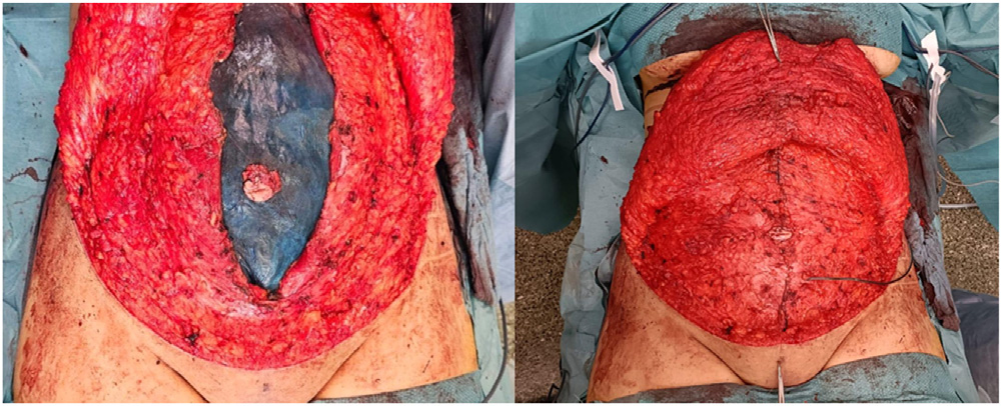
Intra-operative view: dermal autologous mesh after inset (left) and after anterior rectus fascia closure (right).

The patient received intravenous prophylactic Cefuroxim prior to skin incision. A standard abdominoplasty approach was performed. A 30 × 15 cm abdominal dermal flap was harvested, deepithelialized, resected, and defatted using a sharp facelift scissor to remove the subcutaneous adipose tissue, with care to preserve a uniform dermal thickness. Methylene blue dye was used intraoperatively to ensure complete deepithelialization and guide final graft shaping^4^ (Fig. 2). The autologous dermal graft was then positioned in the retro-rectus space (sublay) according to the Rives–Stoppa technique and fixed with interrupted Vycril 2.0 stitches (Ethicon Inc., USA) to the posterior rectus sheath. The anterior rectus fascia was then closed by two overlaps of Maxon 2.0 running sutures (Ethicon Inc., USA) (Fig. 3). After repositioning the abdominal flap, the superficial fascial layer was closed with Vicryl 2.0 (Ethicon Inc., USA). Two subcutaneous drains and one retromuscular drain were placed and secured. The incision was then closed with reversed Monocryl 3.0 suture and completed with a Monocryl 4.0 running intradermal suture (Ethicon Inc, USA). The umbilicus was then repositioned and umbilicoplasty was performed using an inverted-U incision and secured in place using reversed intradermal Monocryl 3.0 suture and Prolene 5.0 Allgöwer-Donati sutures (Ethicon Inc, USA) (Fig. 3). An abdominal compressive garment was applied at the end of the procedure.

**Fig. 4:**
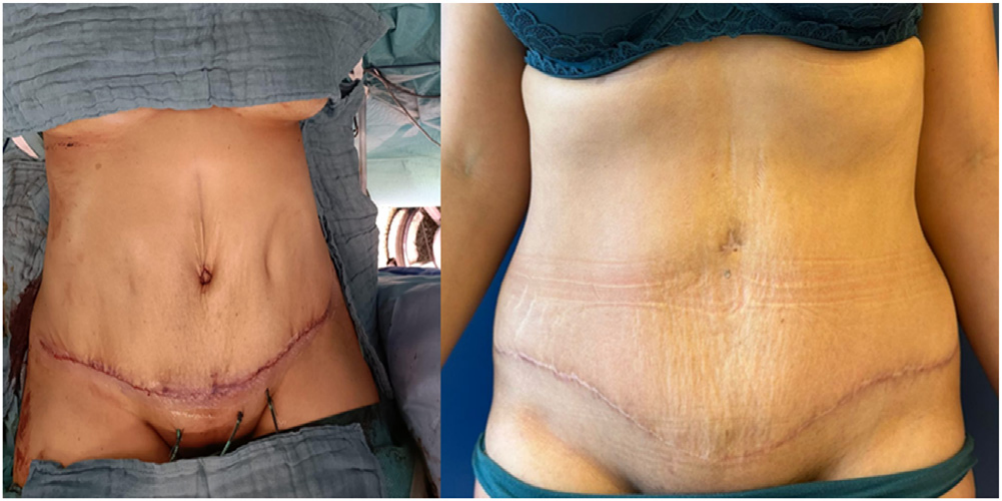
Immediate postoperative view (left), postoperative result at 12-month follow-up (right).

One week postoperatively, the patient required surgical debridement for umbilical necrosis followed by immediate reconstruction using local flaps. The postoperative course thereafter was uncomplicated. The patient declined further umbilical scar revision. At the one-year follow-up, clinical examination and ultrasound imaging showed no recurrence of RD, and the patient expressed high satisfaction with both functional improvement and cosmetic outcome (Fig. 4).

## Discussion

This case highlights the utility of autologous dermis as a biologic reinforcement option for abdominal wall reconstruction in the setting of a giant rectus diastasis with ventral hernia. In the present case, the diastasis was measured at 15 cm intraoperatively and a preoperative CT scan showed a 19 cm RD. Large published case series by Bucaria et al. and Lo Torto et al. report maximum diastasis widths of 8–10 cm.^1^^,^^2^ To the best of our knowledge, this represents the most severe case of rectus diastasis treated with autologous dermal reinforcement reported to date. Similar to Bucaria et al., we placed the autologous dermal mesh in a sublay position, supported by some studies demonstrating lower recurrence rates with retromuscular mesh reinforcement.^5^, ^6^, ^7^

A monocentric retrospective study by Bucaria and Boccuzzi involving 101 patients who underwent autologous dermal mesh reinforcement for severe post-pregnancy rectus diastasis demonstrated that this technique was both safe and effective.^1^ In their series, the authors included patients who had diastasis greater than 6 cm – with a mean RD of 7.29 cm (range 6–10 cm), as assessed by ultrasound evaluation. Lo Torto et al. reported the use of autologous dermal grafts in post-bariatric surgery patients with RD undergoing abdominoplasty. Their series included 24 patients with RD ranging from 4 to 8 cm. After plication of the rectus abdominis muscles, the dermal graft was placed in an onlay position.^2^ No RD recurrence was observed at 2-year follow-up. However, one case of surgical site infection was reported. Previously described by Mutlu et al. in 2014, the use of autologous dermal reinforcement for abdominal wall hernias identified during abdominoplasty was shown to be effective, with magnetic resonance imaging (MRI) confirming the absence of recurrence during follow-up.^3^ Moreover, they demonstrated favorable biomechanical properties, showing that compared to an acellular dermal matrix (FlexHD®, MFT Biologics, USA), dermal autograft showed comparable tensile strength and a higher maximum load before yield. These findings were further supported in a study on the biomechanics of abdominal skin, comparing the maximum tensile strength between abdominal dermis and commercial meshes, and demonstrating that abdominal dermis had a superior tensile strength than commercial meshes.^8^

The occurrence of umbilical necrosis in our patient further emphasizes the advantage of autologous dermal reinforcement. Such a complication could have resulted in a mesh infection, leading to further interventions and mesh removal. The absence of prosthetic material mitigated this risk. While there is a paucity of literature reporting the rate of umbilical necrosis, with many studies reporting umbilical necrosis as a minor wound complication, it is estimated to range between 0.2 – 4.2%.^9^ In the present case, the risk of umbilical necrosis was augmented by the concomitant presence of an umbilical hernia. Although mesh-based repair of ventral hernias has been demonstrated to lower the recurrence rate, the use of prosthetic materials remains controversial due to concerns regarding infection, extrusion and the potential need for mesh removal.^10^ Cost-effectiveness represents an additional advantage of the autologous dermis mesh.

Although current evidence is limited to case reports and small series, outcomes suggest that autologous dermis reinforcement is both safe and effective. In our patient, recovery was satisfactory, with durable abdominal wall support and excellent long-term functional and aesthetic results.

## Conclusion

Autologous dermis reinforcement during abdominoplasty is a feasible and effective option for the repair of severe rectus diastasis. By avoiding prosthetic material, it offers a cost-effective autologous alternative to synthetic mesh, particularly advantageous in patients at risk for wound complications.

## Funding

None.

## Patient consent

The patient provided written consent for the use of anonymized clinical data.

## Declaration of competing interest

Daniel Kalbermatten is Editor in Chief of JPRAS Open and Deputy Editor for the JPRAS, he was not involved in the editorial review or the decision to publish this article.
